# Soft and Robust Identification of Body Fluid Using Fourier Transform Infrared Spectroscopy and Chemometric Strategies for Forensic Analysis

**DOI:** 10.1038/s41598-018-26873-9

**Published:** 2018-05-31

**Authors:** Ayari Takamura, Ken Watanabe, Tomoko Akutsu, Takeaki Ozawa

**Affiliations:** 1 First Department of Forensic Science, National Research Institute of Police Science, 6-3-1, Kashiwanoha, Kashiwa, Chiba 277-0882 Japan; 2 Department of Chemistry, Graduate School of Science, The University of Tokyo, 7-3-1, Hongo, Bunkyo, Tokyo 113-0033 Japan

## Abstract

Body fluid (BF) identification is a critical part of a criminal investigation because of its ability to suggest how the crime was committed and to provide reliable origins of DNA. In contrast to current methods using serological and biochemical techniques, vibrational spectroscopic approaches provide alternative advantages for forensic BF identification, such as non-destructivity and versatility for various BF types and analytical interests. However, unexplored issues remain for its practical application to forensics; for example, a specific BF needs to be discriminated from all other suspicious materials as well as other BFs, and the method should be applicable even to aged BF samples. Herein, we describe an innovative modeling method for discriminating the ATR FT-IR spectra of various BFs, including peripheral blood, saliva, semen, urine and sweat, to meet the practical demands described above. Spectra from unexpected non-BF samples were efficiently excluded as outliers by adopting the Q-statistics technique. The robustness of the models against aged BFs was significantly improved by using the discrimination scheme of a dichotomous classification tree with hierarchical clustering. The present study advances the use of vibrational spectroscopy and a chemometric strategy for forensic BF identification.

## Introduction

Forensic science is an applied research field that aims to develop and validate analytical methods for criminal investigation and justice. Although research interest in forensics is quite diversified, the analysis of biological samples collected at a crime scene is undoubtedly one of the most important tasks owing to the ability to identify criminal acts and suspects by DNA profiling. The identification of body fluid (BF) evidence, such as blood, semen and urine, plays a large role in the analysis of biological samples, as BF evidence is frequently left at crime scenes, and the existence of BFs is often highly suggestive of how the crime was committed^1,2^. Furthermore, BFs can provide reliable origins of DNA^1,3^.

To date, various analytical techniques have been developed to identify BFs^1,2,4^. Most methods used in actual criminal investigations are targeted to detect enzymatic activities or antigens specific to each type of BF. Such methods have been studied and improved for higher specificity, sensitivity and practical ease of handling. However, methods mainly relying on serological and biochemical techniques suffer from several fundamental issues in practice: they consume evidence and are expensive, time consuming and dependent on the judgment of experts. The variety of methods specific to each BF type, which requires the preparation of many experimental reagents and instruments, increases the financial cost. Furthermore, social interest in objective and reliable analytical results has also increased. Thus, novel methods must show non-destructivity, higher cost-performance, greater versatility and objectivity.

Recent developments in BF analysis using vibrational spectroscopy have shown great potential to offer an alternative approach^5–7^. Vibrational spectroscopy, such as Fourier transform infrared spectroscopy (FT-IR) and Raman spectroscopy, has advantageous properties for application to forensic BF identification; they characterize a sample non-invasively using light irradiation, and the spectral patterns are highly selective based on the molecules composing the sample. Detailed peak assignments have shown that BF spectra have characteristic spectral patterns interpretable with physiological knowledge of the components^8–13^. These previous reports indicated the possibility to distinguish BF types based on their spectral patterns. Additionally, the rapid and easy measurement of the spectra is quite advantageous compared to the present bioanalytical methods, which sometimes require complicated experimental protocols or several days for assaying.

One difficulty in identifying BFs by vibrational spectroscopy is the fact that BFs are complex mixtures, which involve donor-specific variations in the number of constituents and inhomogeneous spatial distributions^14^. Therefore, a BF type cannot be identified by a single spectral pattern. Statistical interpretation of the multiple components in the BF spectrum is needed.

Furthermore, some critical problems in its practical application to forensic BF analysis remain. Forensic BF evidence is sometimes subject to undesirable conditions, such as long elapsed time since deposition (or sample aging and degradation) and being mixed with other types of BF and non-BF materials. Moreover, the BF evidence needs to be distinguished from all other suspicious materials. In current serological and biochemical techniques, which detect single target molecules specific to a BF type, sample degradation or the presence of unexpected materials results in weak or no detection of the target molecule. In this way, the results are relatively clear: detected (positive) or not detected (negative). In contrast, the relative intensities of bands in BF spectra can be disturbed by sample degradation and the existence of other materials^8,15–20^. Therefore, careful treatment of the spectra is required for appropriate interpretation. However, the generalization and comprehensive data collection of such spectral disturbances are not feasible. Thus, the development and validation of appropriate techniques using vibrational spectroscopy for such practical forensic issues are scarce.

Herein, to establish the usefulness of vibrational spectroscopic techniques to forensic BF identification, we explored innovative chemometric strategies to overcome the complicated issues described above. Chemometric techniques, or multivariate statistical modeling techniques, have been applied to discriminate BF spectra for various analyses, such as types of BFs^14,21,22^, human versus animal BFs^23,24^ and the donor’s gender^25,26^. Chemometric techniques offer the efficient detection of spectral differences, objective discrimination of BFs with multivariate statistics and ease of analysis even by non-specialist of spectroscopy. The design and detailed algorithm for discriminant modeling are critically important to the discrimination performance of the model for the data of interest. In this study, we describe a novel discriminant modeling method using attenuated total reflection (ATR) FT-IR spectroscopy, considering the practical performance demands in forensic BF identification, such as (1) ability to identify multiple BF types (and availability to increase of the number of BF types to be identified), (2) soft response against unexpected non-BF components and (3) robust response against BF sample aging. To meet these demands, we developed a discrimination scheme incorporating a dichotomous classification tree and Q-statistics testing to exclude unexpected samples as outliers. The developed model demonstrated great discrimination performance towards all tested BF types, aged BFs and unexpected samples, indicating its usefulness in forensic BF analysis.

## Results and Discussion

### ATR FT-IR spectra of fresh body fluid samples

We collected the ATR FT-IR spectra of 5 types of BFs, peripheral blood, saliva, semen, urine and sweat, from ten to twenty Japanese volunteers. The BF types examined are those most frequently collected at crime scenes. As reported previously, BF spectra have spatially dependent variations, as well as donor-dependent variations^14^. Therefore, the ATR FT-IR spectra were collected from different areas in the BF samples dried overnight, resulting in a total of 100 spectra for peripheral blood, semen and urine; 90 spectra for saliva; and 75 spectra for sweat.

**Figure 1:**
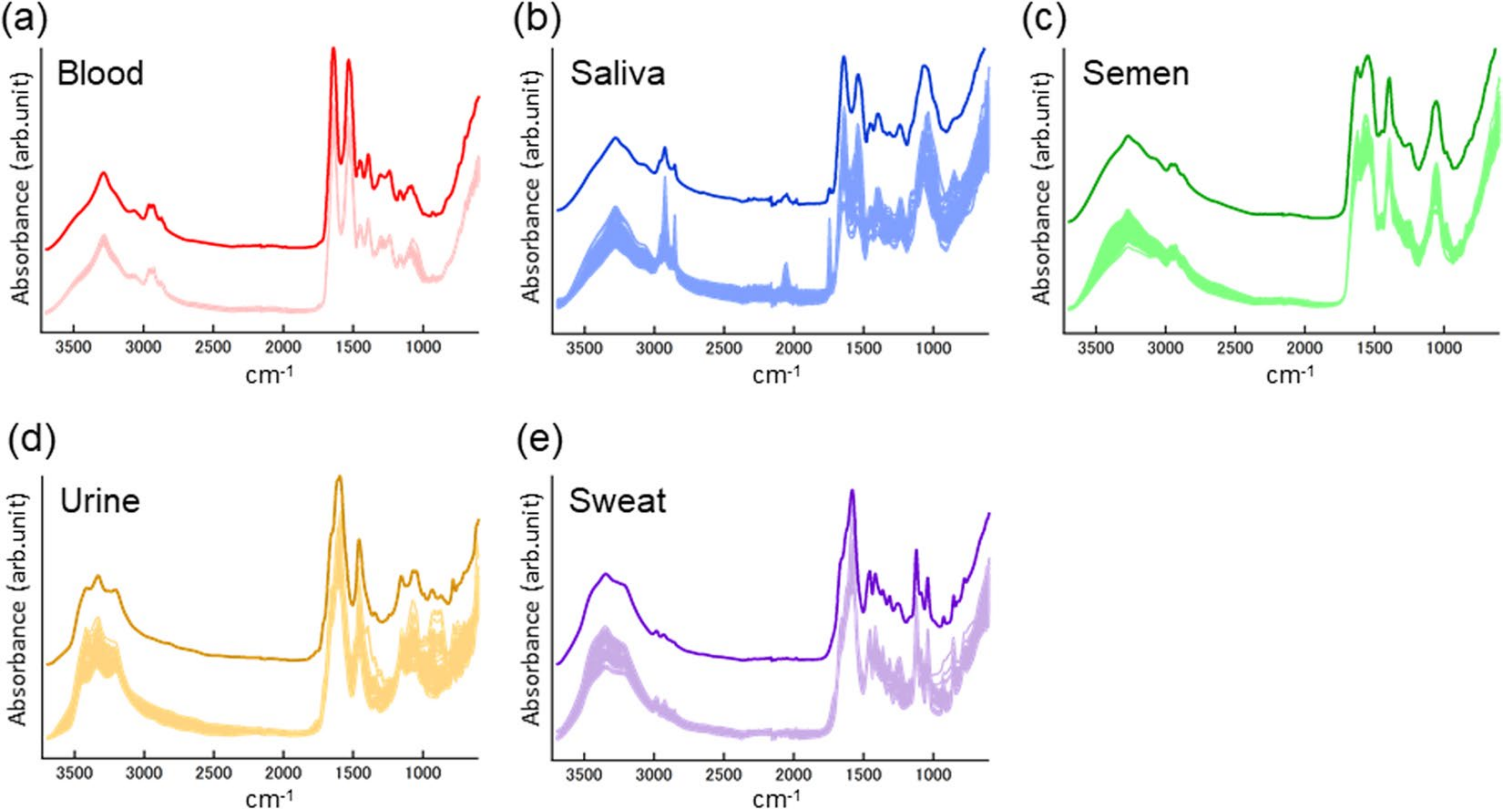
Characterization of the ATR FT-IR spectra of five types of BFs. Collected individual ATR FT-IR spectra (overlaid at the bottom) and the average spectra (top) from peripheral blood (**a**), saliva (**b**), semen (**c**), urine (**d**) and sweat (**e**) dried at ambient conditions overnight. Each spectrum was normalized by the total area.

The collected individual spectra and average spectra of each BF type after normalization by the total area are represented in Fig. 1. The average spectrum of each BF type showed a characteristic pattern, corresponding to previous reports^8,9,27–30^. Dried blood is abundant in hemoglobin from erythrocytes and human serum albumin in plasma. The dominant contributions from such blood proteins correspond to the distinctive peaks in the average spectra of blood at 3285 cm^−1^ (Amide A), 1640 cm^−1^ (Amide I), 1532 cm^−1^ (Amide II) and 1240 cm^−1^ (Amide III). In addition, glucose represents the contribution in the spectral range of 1100-980 cm^−1^, which shows substantial donor variance^8,9,24,28^. The average spectrum of saliva also shows characteristic peaks of proteins at 3279 cm^−1^ (Amide A), 1640 cm^−1^ (Amide I), 1537 cm^−1^ (Amide II) and 1239 cm^−1^ (Amide III)^30–32^. The existence of thiocyanate anions (SCN^−^) is reflected by the typical peak at 2057 cm^−1^ ^9,31^. Lipids in oral mucosa correspond to the peaks at 2943 cm^−1^, 2854 cm^−1^ and 1743 cm^−1^, which results from the symmetric and asymmetric C–H stretching and carbonyl (C=O) stretching of the ester groups, respectively^33,34^. Sugar moieties of glycosylated proteins, including α-amylase, show large variances in the spectral range of 1080-950 cm^−1^ ^9,31^. Abundant proteins in semen, such as albumin and acid phosphatase, dominantly contribute the strong peaks observed at 3268 cm^−1^ (Amide A), 1622 cm^−1^ (Amide I), and 1547 cm^−1^ (Amide II). The distinctive peak at 1056 cm^−1^ can be assigned to contributions from the sugar moieties of prostate specific antigen and fructose, which are abundant in human semen. The peak observed at 1393 cm^−1^ originates from the methyl groups of proteins and lipids^9,28,34^. Meanwhile, the average spectra of urine and sweat indicate little contribution from proteins. Urea is a major component in urine, and distinctive peaks for urea at 1592 cm^−1^ (C=O stretching), 1457 cm^−1^ (C-N stretching) and 1154 cm^−1^ (NH_2_ deformation) are found in the average spectrum of urine^27,35^. Small contributions from creatinine, another typical component of urine, were found as weak peaks at 1334 cm^−1^ and 1237 cm^−1^ ^36^. The sweat samples contain lactate as a dominant molecule, which exhibits strong signals at 1580 cm^−1^, 1416 cm^−1^, 1121 cm^−1^ and 1040 cm^−1^ ^29^. Additionally, urea also shows a distinguishable contribution at 1455 cm^−1^. Although these BF samples contain some common constituents, the variety of types and relative amounts of constituents can distinguish the spectral patterns of the BFs, even by visual comparison.

In addition, the BF spectra involved substantial variations dependent on the sample donor and spatial distribution of the sample (Fig. 1). The trends of such spectral variations in the types of BFs are important to understand. Principle component analysis (PCA) of the spectral dataset provides a relative evaluation of the variances of the dominant spectral components, called principle components (PCs). A set of ATR FT-IR spectra for each BF type was mean centered and subsequently applied to PCA. Figure S-1 shows the calculated scores of the 1st and 2nd PCs for each set of BF spectra. The spread of the score plots almost corresponds to the spectral variations among the BF data. The degree of spectral variance was substantially different among BF types and was the smallest in blood and the largest in saliva and urine. This trend is consistent with the visual comparison of the various individual spectral patterns of each type of BF, overlaid in Fig. 1. Furthermore, relative variances among all the collected BF spectra were also assessed by PCA (Fig. S-2). The spectra from each BF type were shown with characteristic contributions from each PC and different spreads of the distributions. These results indicated that the spectral characteristics of a BF type cannot be represented by a single or averaged spectrum. Multivariate statistical analysis of the spectral variations is essential to characterize and objectively distinguish the BF spectra.

### Multi-class classification of fresh body fluid spectra

**Figure 2:**
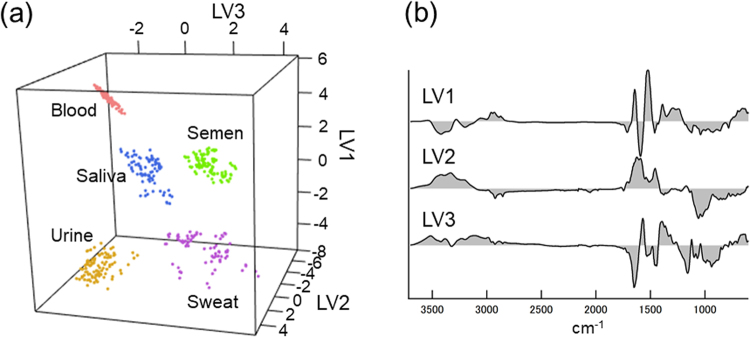
Five-class PLS-DA modeling for the ATR FT-IR spectra of fresh BFs. (**a**) A three-dimensional dot plot of the PLS-DA scores for the ATR FT-IR spectra of five BF types, peripheral blood (pink), saliva (blue), semen (light green), urine (yellow) and sweat (light purple), calculated by the five-class PLS-DA model for fresh BFs. (**b**) Weight loading vectors corresponding to the first three LVs in the five-class PLS-DA model.

We used a multivariate discriminant analysis method, partial least squares discriminant analysis (PLS-DA), to discriminate the ATR FT-IR spectra of various BFs with statistical consideration of the spectral variances. The PLS-DA algorithm re-plots the spectral data in an alternative subspace on latent variables (LVs) so that the data of each group are distributed separately. The collected spectra of five BF types, 100 blood spectra, 90 saliva spectra, 100 semen spectra, 100 urine spectra and 75 sweat spectra, were examined by the PLS-DA algorithm. Then, a five-class PLS-DA model was constructed using four LVs by 5-fold cross validation (CV) using spectral data from independent donors for calibration and testing datasets, respectively. The calculated PLS-DA scores of each spectral data set for the first three LVs are represented in Fig. 2a. Each BF group was efficiently segregated in this subspace, and variances within the groups were also exhibited. Although the chemical meaning of each LV is difficult to precisely interpret, we supposed that the first LV indicated the positive contribution from proteins with Amide I and Amide II and the negative contribution from urea C=O stretching at 1590 cm^−1^ (Fig. 2b). The second LV contained a distinctive signal from sugar moieties at approximately 1060 cm^−1^, which resulted in the characterization and segregation of saliva and semen. The third LV showed typical signals for lactate at 1121 cm^−1^ and 1040 cm^-1^, which mainly contributed to the discrimination between urine and sweat. Subsequently, the PLS-DA scores for the four LVs were applied to the linear discriminant analysis (LDA) algorithm to provide a discriminant model for final assignment of the data into one of the BF groups. As a result, all 475 BF spectral data points observed in this study were correctly classified into their proper BF category.

### Soft response against unexpected samples using the Q-statistics technique

**Figure 3:**
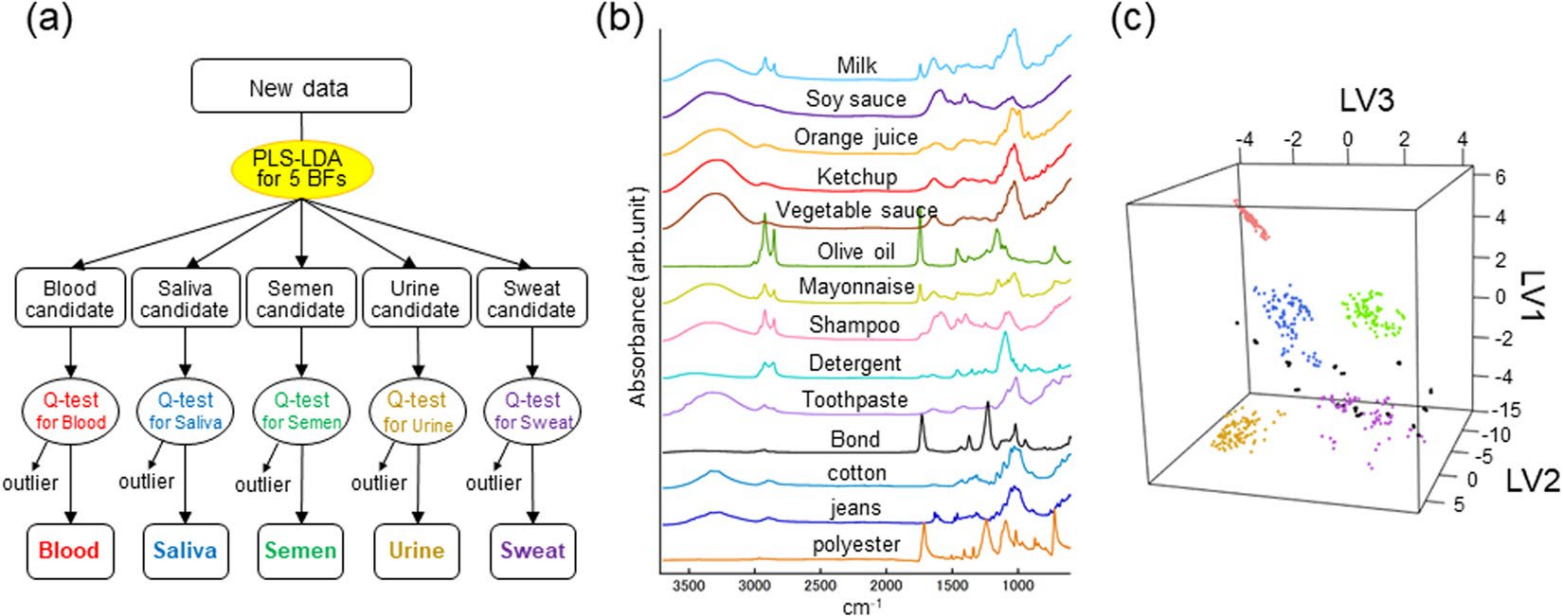
Identification of the ATR FT-IR spectra of non-BF samples as outliers using Q-statistics for each BF group. (**a**) A discrimination scheme using a PLS-LDA model for the five BF types followed by Q-tests for each BF type. (**b**) Average ATR FT-IR spectra of prepared non-BF samples, including milk, soy sauce, ketchup, vegetable sauce, olive oil, mayonnaise, shampoo, laundry detergent, toothpaste, glue, cotton cloth, jeans and polyester cloth. All spectra were normalized by the total area. (**c**) PLS-DA scores for the ATR FT-IR spectra of the non-BF samples, predicted by the five-class PLS-DA model for fresh BFs (black dots).

The PLS-DA model incorporating the LDA algorithm (the PLS-LDA model) showed great performance with high discrimination accuracy for the spectral data of five BFs. However, both PLS-DA and LDA are hard modeling methods, which were designed to assign each data point into one of the modeled classes. This means that the model cannot identify non-BF or unexpected samples and may possibly misclassify them as one of the modeled BFs. This property is undesirable in forensic analysis because a variety of suspicious materials can be encountered in criminal investigation, including foods and chemical detergents, as well as BFs. To overcome this problem, we proposed a novel discrimination scheme, a PLS-LDA model followed by Q-statistics tests, for each BF class (Fig. 3a). Q-statistics is a one-class classification technique to determine whether a data point belongs to a certain class and is known as a soft modeling method^37–39^. The suitable feature of Q-statistics for spectral discrimination is the evaluation of the sum of residuals after spectral regression. The PLS-DA algorithm comprises a sequence of regression of the spectral data to LV vectors, providing the corresponding PLS-DA scores and residuals. While PLS-DA scores are used to indicate the group to which the data belong, the residuals correspond to how well the data are explained by the model. Therefore, if the examined spectral data does not correspond to one of the modeled-BFs, the sum of residuals after PLS-DA spectral regression is significantly larger than that of the modeled BF, and the data is determined to be an outlier using a threshold value. To demonstrate the usability of this alternative discrimination scheme, we prepared fourteen types of non-BF samples, including milk, soy sauce, orange juice, ketchup, vegetable sauce, olive oil, mayonnaise, shampoo, laundry detergent, tooth paste, glue, cotton cloth, denim and polyester cloth, as examples of unexpected samples. Five spectra were collected from each of the fourteen non-BF samples (Fig. 3b), resulting in a total of 70 spectra. Then, both the BF spectra and non-BF spectra were subjected to the PLS-LDA model for the five BFs (Fig. 3c, Table S-1) and subsequently examined by Q-tests for the corresponding type of BF built with 5-fold CV. Table 1 shows the discriminant result using a threshold of 99.5% statistical significance for the Q-tests. All non-BF spectra were successfully excluded as outliers. In addition, almost all the BF spectra were assigned to the correct BF group. Therefore, the proposed discrimination scheme, combining hard (PLS-DA and LDA) and soft (Q-statistics) modeling methods, affords strong discrimination performance for the target BFs and simultaneously offers a soft response to exclude unexpected data points as outliers. This modeling strategy provides suitable applicability to forensic investigations in which BFs need to be identified from various suspicious materials found at crime scenes.

**Table 1:** Discrimination results for the ATR FT-IR spectra of fresh BFs and unexpected samples using the five-class PLS-LDA model followed by Q-tests for each BF group validated.

Actual Body Fluid Type	Predicted Body Fluid Type					
	Blood	Saliva	Semen	Urine	Sweat	Outlier^a^
Blood	100	0	0	0	0	0
Saliva	0	89	0	0	0	1
Semen	0	0	99	0	0	1
Urine	0	0	0	97	0	3
Sweat	0	0	0	0	74	1
Non-BF	0	0	0	0	0	70

^a^Q-tests for each BF group were implemented with a statistical significance level of 99.5%.

### Multi-class classification of aged body fluid spectra

**Figure 4:**
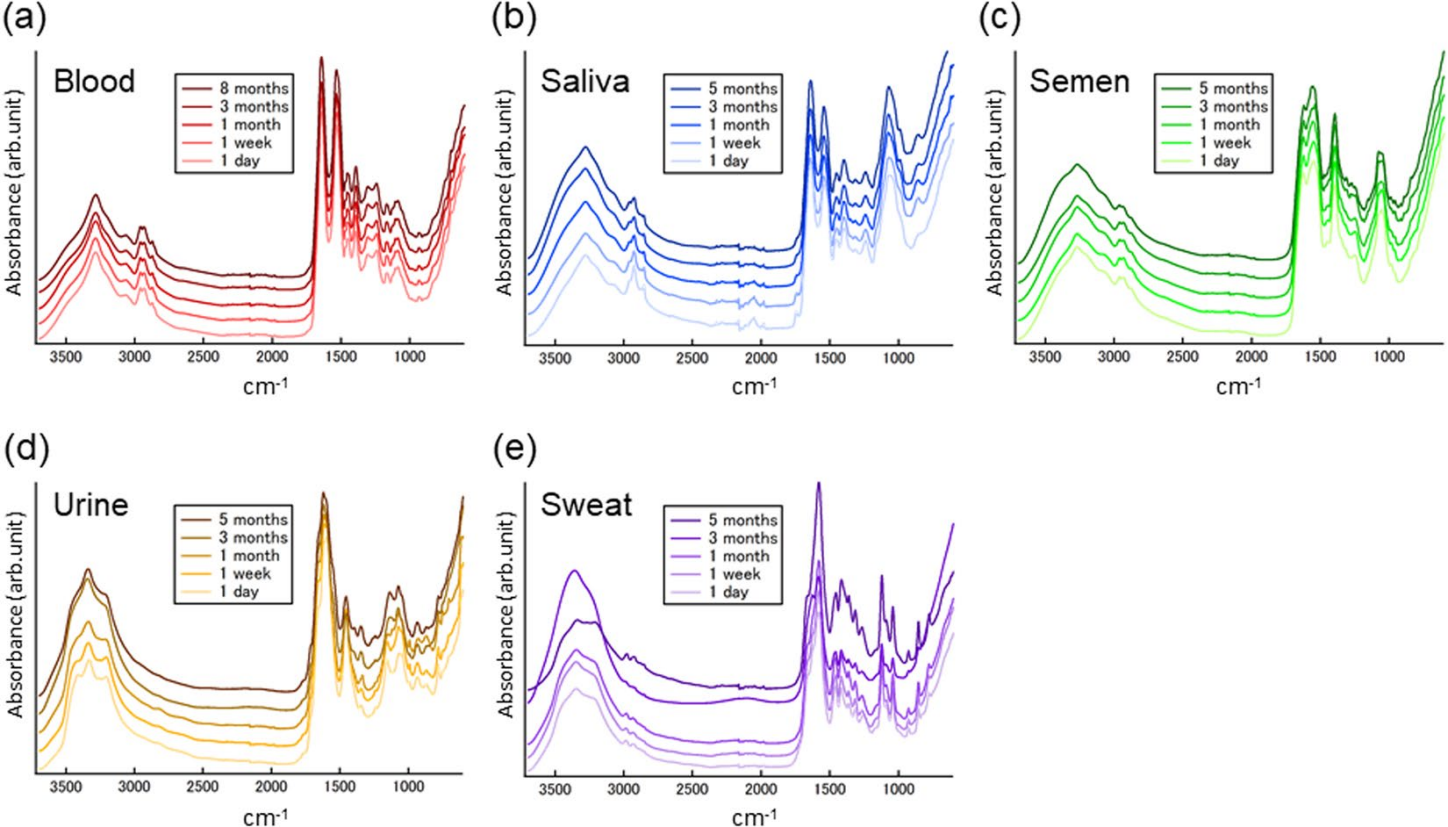
Spectral changes in the five BF types with aging over several months. Average ATR FT-IR spectra of peripheral blood (**a**), saliva (**b**), semen (**c**), urine (**d**) and sweat (**e**) aged from one day to eight months. The lighter color spectra indicate those of fresher BF samples. The darker color spectra correspond to those of aged BF samples. The ages of the BF samples are given in each graph.

The other concern in this study is the applicability of the discriminant methods to aged BF samples. BF samples may be collected long after deposition or the occurrence of the crime. To assess the spectral changes with sample aging, we collected ATR FT-IR spectra of aged BF samples stored at ambient conditions for one day, one week, one month, three months, five months or eight months (Fig. 4). In addition, to enhance our understanding of the spectral changes, difference spectra of the aged BF spectra from the fresh (1 day) BF spectra were also obtained (Fig. S-3). Compared to the standard deviation spectra of the 1-day BFs, significant spectral disturbances were observed for each type of BF after aging of more than one month. Although it is difficult to interpret all the spectral changes in detail, decreases in the characteristic bands of proteins, Amide I and Amide II were identified in the spectra of aged blood, saliva and semen, indicating degradation of the protein structures. The spectra of aged urine and sweat showed possible influences from differences in humidity, or water content, in the 3500-3100 cm^−1^ region. However, significant decreases in the signals at approximately 1590 cm^−1^ and 1460 cm^−1^ in the aged urine spectra and approximately 1455 cm^−1^ in the aged sweat spectra were indicative of the decomposition of urea. PCA for all the aged BF spectra showed more diffused and overlapped distribution of the spectral data of each BF type than that of 1-day BF spectra (Fig. S-4). This indicates that the spectral dataset was more complicated and contained various spectral components.

The collected spectra of the aged BFs were first subjected to the five-class PLS-LDA model (Table S-2) and subsequently applied to the corresponding Q-tests, constructed using the above spectral data for the 1-day BFs (designated the “1day-5class PLS-LDA-Q model”) (Table S-3). The five-class PLS-LDA model showed relatively good performance for discriminating the spectra of fresher BFs, including those aged from 1 day to 1 month. However, upon further aging of the BF samples, some of the spectra were misclassified into the incorrect BF groups. The insertion of the Q-tests in the 1day-5class PLS-LDA-Q model were effective to exclude some misclassified data as outliers. But the other misclassified data were still remained in the incorrect BF groups, and simultaneously some data correctly assigned by the five-class PLS-LDA were removed into outliers, resulted in false-negatives. Moreover, the PLS-DA scores calculated for the aged BF spectra showed shifts and spreads of the distribution for each type of BF, indicating the poor discrimination performance of the model (Fig. S-5A). Consequently, the 1day-5class PLS-LDA-Q model was not useful to classify the spectra of aged BFs.

To investigate modeling strategies for improving the discrimination performance against the aged BF spectra, we built an alternative five-class PLS-LDA-Q models by incorporating details about the aged BF spectra from one day to eight months by 5-fold CV using five LVs (designated the “aged-5class PLS-LDA-Q model”) (Fig. S-5B). Because this model learned variances among the observed aged BF spectra, all the aged BF spectra were correctly classified by the preliminary PLS-LDA step, and finally the great discrimination accuracy was demonstrated, independent of ages of BF samples (Table S-4). However, the statistical significance level for Q-tests to eliminate unexpected data decreased to 99.0% compared to that of the 1day-5class PLS-LDA-Q model (99.5%). In addition, the number of LVs required for the model increased. This means that the regression efficiency of the model for the spectral data decreased. Thus, the model has lower sensitivity to detect outliers. Furthermore, the experimental cost to construct the model is quite high, as many spectra of aged BFs must be collected over several months as training data. Nevertheless, the applicability to BF spectra of unexamined ages, such as intermediately aged or further aged, is not assured. Therefore, the aged-5class PLS-LDA-Q model was also regarded as not suitable for a practical forensic methodology to identify BFs.

### Robust response against sample-aging using a dichotomist classification tree

Considering the results obtained above, we developed a hypothesis to describe possible difficulties in multi-class classification using the PLS-DA algorithm; to discriminate multi-class data, an alternative subspace on LVs can be separated into multi-regions specific to the scores of each class. As the number of classes to be discriminated increases, the characterization efficiency by an axis decreases per class. Thus, more dimensions are required to separate all the classes, resulting in a more complex model structure. Such complex model is more vulnerable to un-modeled fluctuations and simultaneously less sensitive to outliers. Therefore, our hypothesis is that the fewer classes, the more efficient and robust the model.

**Figure 5:**
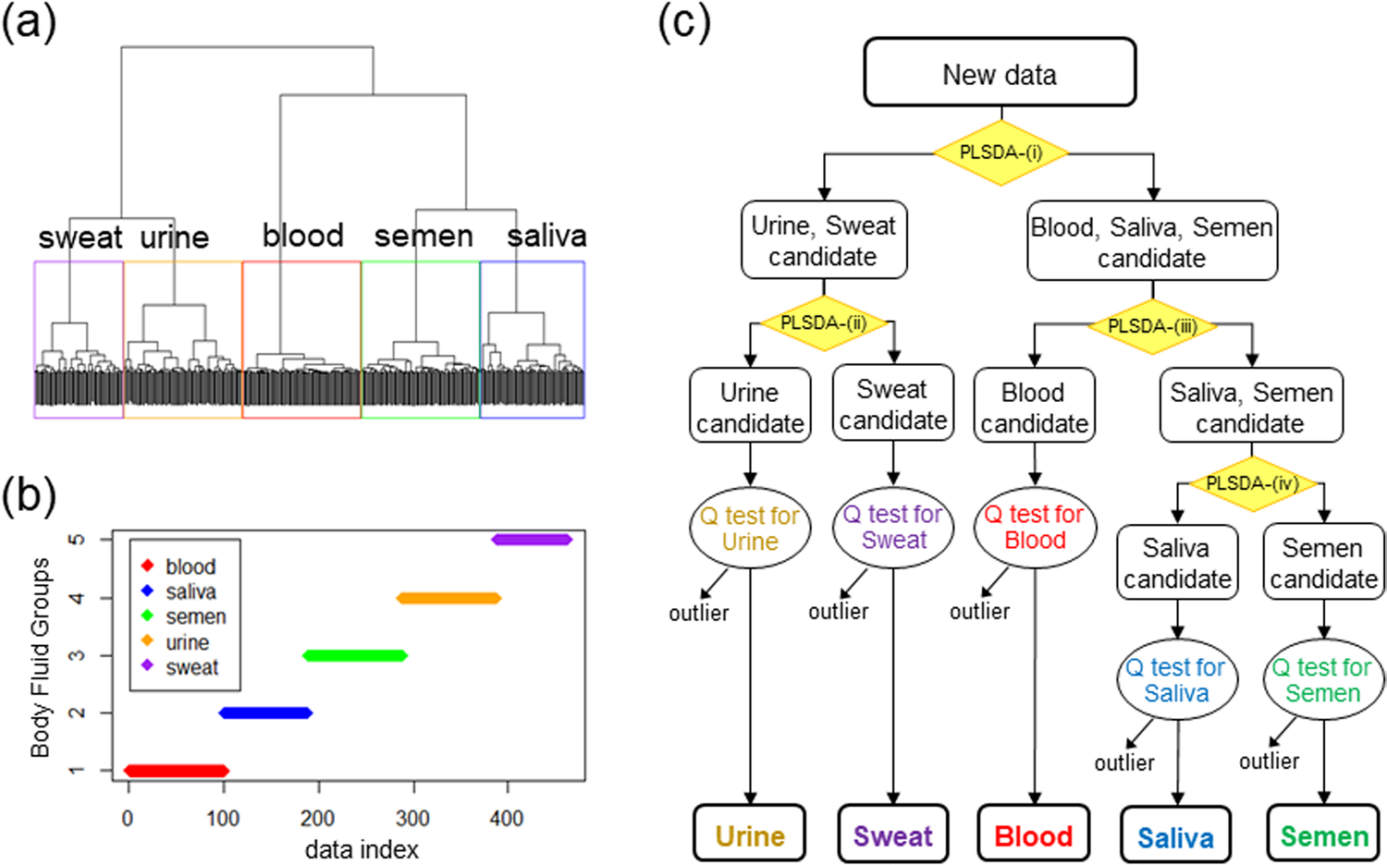
Development of a dichotomous classification tree for the ATR FT-IR spectra of five BF types based on hierarchical clustering analysis. (**a**) A dendrogram established for the PLS-DA scores of 1-day BF spectra predicted by the 1day-5class PLS-LDA-Q model. Borders drawn in the dendrogram indicate clusters of data from each BF group, peripheral blood (red), saliva (blue), semen (green), urine (orange) and sweat (purple). (**b**) Assignment of the data from each spectrum into the respective BF group. (**c**) A discrimination scheme using a dichotomous classification tree followed by Q-tests for each type of BF. The dichotomous classification tree was built according to the dendrogram. Four 2-class PLS-DA models for the corresponding BF groups were set at each node of the tree: (i) blood-saliva-semen versus urine-sweat using four LVs, (ii) urine versus sweat using four LVs, (iii) blood versus saliva-semen using two LVs and (iv) saliva versus semen using three LVs.

To incorporate this idea into our discriminant modeling strategy, we performed the hierarchical clustering analysis of the PLS-DA scores of the 1-day BF spectra predicted by the 1day-5class PLS-LDA-Q model. Hierarchical clustering enables evaluation of the similarities between data and organizes the clusters in a hierarchy. As a result, the PLS-DA scores of the 1-day BF spectra were perfectly clustered into each BF group, corresponding to the discrimination result above (Fig. 5a,b). The established dendrogram showed four spots in which the pairs of BF clusters merge: (1) saliva and semen, (2) blood and saliva-semen, (3) urine and sweat, and (4) blood-saliva-semen and urine-sweat. According to the structure of the dendrogram, a novel discriminant scheme was built, consisting of a dichotomous classification tree and Q-tests inserted at each end (Fig. 5c). The dichotomous classification tree equips 2-class PLS-DA models for the corresponding BF groups at each node, constructed with only 1-day BF spectra by 5-fold CV. The spectra of the aged BFs and unexpected samples were applied to the novel discriminant scheme (Table 2). Consequently, most of the aged BF spectra were classified into the correct BF group, indicating the great robustness of the discriminant scheme against spectral disturbances with sample aging. In addition, the statistical significance level for Q-tests to exclude unexpected data was adequately high. Therefore, the 2-class PLS-DA algorithm enabled characterization of the spectral data of each BF group quite efficiently. The discrimination performance against aged BFs and unexpected samples was drastically improved by the novel modeling strategy using a dichotomous classification tree and Q-statistics over that by multi-class modeling.

**Table 2:** Discrimination results for the ATR FT-IR spectra of aged BFs (1 day to 8 months) and unexpected samples using the dichotomous classification tree followed by Q-tests for each BF group.

Actual Body Fluid Type		Predicted Body Fluid Type					
		Blood	Saliva	Semen	Urine	Sweat	Outlier^a^
Blood	1 day	100	0	0	0	0	0
	1 week	100	0	0	0	0	0
	1 month	100	0	0	0	0	0
	3 months	98	0	0	0	0	2
	8 months	98	0	0	0	0	2
Saliva	1 day	0	90	0	0	0	0
	1 week	0	90	0	0	0	0
	1 month	0	88	0	0	0	2
	3 months	0	90	0	0	0	0
	5 months	0	85	0	0	0	5
Semen	1 day	0	0	100	0	0	0
	1 week	0	0	100	0	0	0
	1 month	0	0	100	0	0	0
	3 months	0	0	100	0	0	0
	5 months	0	0	100	0	0	0
Urine	1 day	0	0	0	100	0	0
	1 week	0	0	0	91	0	9
	1 month	0	0	0	99	0	1
	3 months	0	0	0	99	0	1
	5 months	0	0	0	95	0	5
Sweat	1 day	0	0	0	0	75	0
	1 week	0	0	0	0	75	0
	1 month	0	0	0	0	73	2
	3 months	0	0	0	0	66	9
	5 months	0	0	0	0	52	23
Non-BF		0	0	0	0	0	70

^a^Q-tests for each BF group were implemented with a statistical significance level of 99.995%.

The modeling strategy of building a dichotomous classification tree based on hierarchical clustering could potentially be used to discriminate more than the five BF groups tested in the present study. Such extendibility is superior to multi-class classification. Moreover, the discrimination accuracy can be further improved by optimizing the 2-class discriminant models at each node, such as by selection of the spectral range, the use of other discriminant algorithms and learning from a BF test set involving spatial and donor variations and sample aging. In addition, the determination of thresholds for the Q-tests can be optimized by further investigation, including applications to more types of non-BF samples and detailed analysis about spectral similarities, increasing reliability for practical use. Furthermore, BF samples absorbed in substrates such as fabrics are sometimes collected in crime scene. In such cases, signals from the substrates interfere in ATR FT-IR spectra of the BF stain. Consequently sophisticated spectral processing is necessary to remove the interfering signals, which was demonstrated in our previous study about bloodstains on various substrates^40^. Further studies about BF stains on interfering substrates will enhance practical usefulness of this discriminant technique in forensic investigation.

In conclusion, we comprehensively studied the ATR FT-IR spectra of various BFs and developed a novel discrimination scheme for multiple BF types that showed softness against unexpected samples and robustness against BF aging. This approach enables critical issues to be overcome for the practical application of vibrational spectroscopy to forensic BF identification. Additionally, this approach offers potential for the number of BF types that can be identified to be expanded and the discrimination accuracy to be increased by further model optimization. Consequently, the present study can contribute to the establishment of a non-destructive and objective methodology of BF identification by vibrational spectroscopy that meets forensic requirements.

Furthermore, the discrimination modeling strategy provided in this study is expected to be useful not only for forensic BF analysis but for various scientific analyses to discriminate multiple groups using multivariate parameters involving variances and undefined fluctuations. The present study may contribute to a universal approach to construct multi-class classification models targeting various complex interests.

## Materials and Methods

### Sample collection and preparation

All procedures involving human participants were carried out in accordance with the guidelines of the National Research Institute of Police Science and approved by the Institutional Ethics Committee of the National Research Institute of Police Science (Kashiwa, Japan). Blood, saliva, semen, urine and sweat samples were collected from ten (peripheral blood), fifteen (sweat), eighteen (saliva) or twenty (semen and urine) healthy Japanese volunteers. Informed written consent was obtained from all BF donors. Blood samples were immediately tested after collection without the addition of anticoagulant. The other BF samples were stored at −80°C until use. 10−30 µL aliquots of each of the collected BF samples were deposited on glass slides and stored at ambient conditions for one day, one week, one month, three months, five months or eight months. Dried components in each BF sample were visually observed and covered the glass slide adequately for the subsequent spectra measurement.

Fourteen types of non-BF samples were prepared, including milk, soy sauce, orange juice, ketchup, vegetable sauce, olive oil, mayonnaise, shampoo, laundry detergent, tooth paste, glue, cotton cloth, denim and polyester cloth. The liquid samples (milk, soy sauce, orange juice, ketchup, vegetable sauce, olive oil, mayonnaise, shampoo, laundry detergent, tooth paste and glue) were deposited on glass slides and stored at ambient conditions overnight. Untreated regions of the three cloth samples (cotton, denim and polyester) were cut into pieces of a few square centimeters.

### Instrumentation and spectra collection

The ATR FT-IR spectra of the BF and non-BF samples were recorded using an FT-IR spectrometer (Spectrum One; PerkinElmer Inc., MA) equipped with an ATR accessory with a ZnSe crystal. The wavenumber range was 600–3700 cm^−1^. The spectral resolution was 4 cm^−1^. Each spectrum was recorded as the average of four scans. The ATR crystal was cleaned with 70% ethanol, and the background spectrum was recorded before every sample analysis. Ten (peripheral blood) or five (the other BFs) spectra were collected from different points on the deposits of each BF sample at each storage span. Five spectra were recorded from different spots on the deposits of each liquid non-BF sample and on pieces of each cloth sample. All the collected spectra were not intervened by the glass signal. The instruments were operated using the included software (Spectrum ver. 5.0.1; PerkinElmer Inc., MA).

### Spectral analysis

All of the collected spectra were preprocessed using IGOR Pro software (WaveMetrics Inc., OR). The spectra were transformed into absorption via a log(1/T) function and subsequently normalized by the total area.

Multivariate analysis of the preprocessed spectra was executed using R software with the Rstudio environment. PCA of the spectral data was performed using the prcomp function in the R package “stats”. Prior to the construction of discriminant models, the preprocessed dataset from 83 donors was randomly split into 5 subsets for 5-fold CV of the models, using the cvsegments function in the R package “pls”. One of the split subsets was selected for testing dataset, and the remaining 4 subsets were combined and assigned to training dataset. A set of 5 spectra from a unique donor was sorted into either of the datasets. A 1-day classification model was built using the 1-day BF spectra of donors of training dataset. Then, the spectra of the other donors for testing dataset, including 1-day to aged BFs, were predicted by the model. To build the aged-5class PLS-LDA-Q model, the 1-day to aged BF spectra of donors for training dataset was used. Subsequently, the 1-day to aged BF spectra from donors for testing dataset were predicted by the model. All of the constructed models were validated by the independent spectral data from the different donors than those used in the training dataset.

To construct classification models for the BF spectra, the PLS-DA algorithm was first implemented using the plsda function in the R package “caret”. PLS-DA scores and response values of each spectral data set were predicted by the sequential projection of the spectral data on weight vectors corresponding to the LVs defined in the PLS-DA model. The ideal number of LVs adopted in the PLS-DA model was determined according to the following criteria:

(i) PRESS(*k*)/RSS(*k-1*) < 0.8
(ii) Explained variance of response values (*k*) > 1%

where *k* indicates the ordinal number of LVs in the PLS-DA model. PRESS(*k*) represents the predicted residual error of the sum of squares of the response values, calculated by 5-fold CV of the PLS-DA model with *k* LVs. RSS(*k-1*) is the residual sum of squares of the response values in the PLS-DA model with *k-1* LVs. If both (i) and (ii) were satisfied, the *k*-th LV was adopted in the model. Note that in the case of a multi-class (five classes in this study) classification model, PRESS, RSS and the explained variance were evaluated for each class individually. Then, if both (i) and (ii) were satisfied in more than half of the classes, the *k*-th LV was adopted.

Subsequently, LDA models were built using the PLS-DA scores via the lda function in the R package “MASS”. The LDA models provide the final assignment of the spectral data to one of the BF types.

Q-statistics is a one-class classification technique which was introduced to exclude spectra of non-BFs or unexpected samples as outliers^37^. The Q^2^ values were calculated with the following formula:

1 $${{Q}}^{2}=\sum _{{\boldsymbol{i}}}{({{x}}_{{observed},{i}}-{{x}}_{{PLSDA}-{regressed},{i}})}^{2}=\sum _{{i}}{{e}}_{{\boldsymbol{i}}}^{2}$$

where ***x*** indicates a vector of a spectrum with length *i*. The Q^2^ values correspond to the sum of residuals of the spectrum after PLS-DA regression^37–39^. A threshold value for Q^2^ with a statistical significance level of α was determined by the following formula:

2 $${{Q}}_{{threshold},\,{\alpha }}^{2}={{\theta }}_{1}{[1+{{\theta }}_{2}{{h}}_{0}(\frac{{{h}}_{0}-1}{{{\theta }}_{1}^{2}})+\frac{{{z}}_{{\alpha }}\sqrt{(2{{\theta }}_{2}{{h}}_{0}^{2})}}{{{\theta }}_{1}}]}^{1/{{\boldsymbol{h}}}_{0}}$$

A detailed explanation of the definition of each parameter in equation (2) can be found elsewhere^37,38^. Q^2^ values larger than the threshold were identified as outliers.

The hierarchical clustering technique was performed to compare similarities among spectra of the five types of BFs prior to the construction of a dichotomist classification tree. The dist function in the R package “stats” was used to evaluate the Euclidean distances between the PLS-DA scores of the 1-day BF data predicted in a 5-class PLS-DA model constructed with only 1-day BF data. Then, the clusters were established using the hclust function with Ward’s criterion^41,42^.

## Electronic supplementary material

Supplementary Information
