# Supplementary material for: Soft and Robust Identification of Body Fluid Using Fourier Transform Infrared Spectroscopy and Chemometric Strategies for Forensic Analysis
Source: Sci Rep. 2018 May 31;8:8459. doi: 10.1038/s41598-018-26873-9 (PMC5981217; doi:10.1038/s41598-018-26873-9)
Supplement: Supplementary file 1 — Supplementary Information [file 41598_2018_26873_MOESM1_ESM.docx]

*Supplementary Information*

**Soft and Robust Identification of Body Fluid Using Fourier Transform Infrared Spectroscopy and Chemometric Strategies for Forensic Analysis**

Ayari Takamura^1, 2*^, Ken Watanabe^1^, Tomoko Akutsu^1^, Takeaki Ozawa^2^^**^

^1^ First Department of Forensic Science, National Research Institute of Police Science

6-3-1, Kashiwanoha, Kashiwa, Chiba 277-0882, Japan

^2^ Department of Chemistry, Graduate School of Science, The University of Tokyo

7-3-1, Hongo, Bunkyo, Tokyo 113-0033, Japan

Correspondence should be addressed to:

Ayari Takamura (*)

E-mail: [takamura@nrips.go.jp](mailto:takamura@nrips.go.jp)

Tel.: +81-4-7135-8001, Fax: +81-4-7133-9159

Takeaki Ozawa (**)

E-mail: ozawa@chem.s.u-tokyo.ac.jp

Tel.: +81-3-5841-4351, Fax: +81-3-5802-2989

**Table of contents**

Figure S-1. Spectral variances among the ATR FT-IR spectra of each BF type evaluated by PCA.S-3

Figure S-2. Spectral variances among all the ATR FT-IR spectra of BFs evaluated by PCA.S-4

Figure S-3. Difference ATR FT-IR spectra of aged BFs compared to fresh BFs.S-5

Figure S-4. Spectral variances among the ATR FT-IR spectra of aged BFs evaluated by PCA.S-6

Figure S-5. Discrimination of the ATR FT-IR spectra of aged BFs using multi-class classification methods. S-7

Table S-1. Discrimination results for the ATR FT-IR spectra of non-BF samples using the PLS-LDA model built with 1-day BF spectra.S-8

Table S-2. Discrimination results for the ATR FT-IR spectra of aged BFs (1 day to 8 months) and unexpected samples using the 1day-5class PLS-LDA model.S-9

Table S-3. Discrimination results for the ATR FT-IR spectra of aged BFs (1 day to 8 months) and unexpected samples using the 1day-5class PLS-LDA-Q model.S-10

Table S-4. Discrimination results for the ATR FT-IR spectra of aged BFs (1 day to 8 months) and unexpected samples using the aged-5class PLS-LDA-Q model. S-11


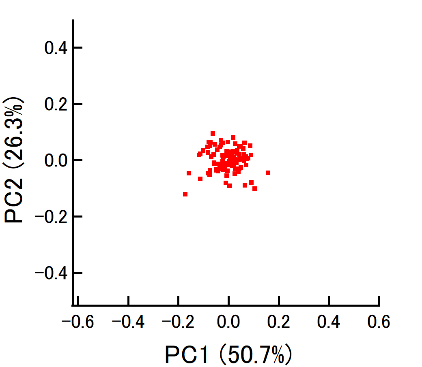


(a)

Blood


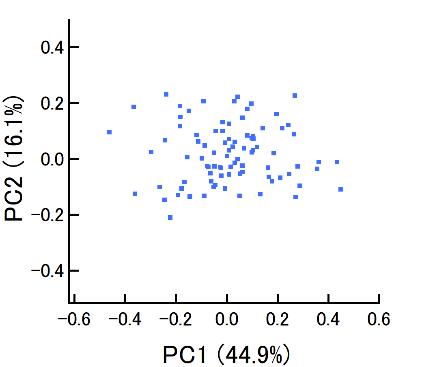


(b)

Saliva


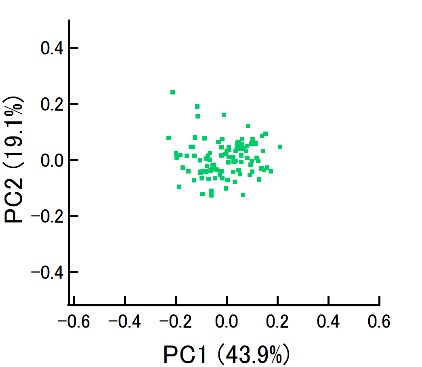


(c)

Semen


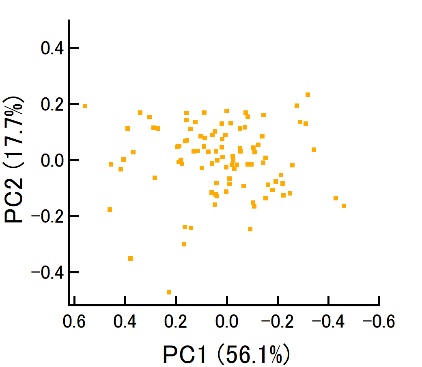


(d)

Urine


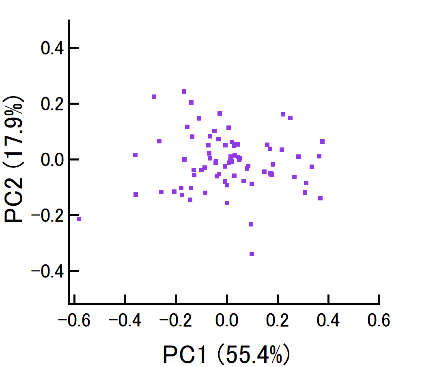


(e)

Sweat

**Figure S-1. Spectral variances among the ATR FT-IR spectra of each BF type evaluated by PCA.** Dot plots of the calculated scores of 1st PC and 2nd PC for the ATR FT-IR spectra of peripheral blood (a), saliva (b), semen (c), urine (d) and sweat (e).


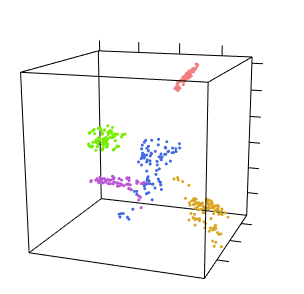


4

2

0

-2

-6

-4

-2

0

2

4

-5

0

5

PC1 (43.6%)

PC2 (30.8%)

PC3 (11.6%)

**Figure S-2. Spectral variances among all the ATR FT-IR spectra of BFs evaluated by PCA.** A three dimensional dot plot of the calculated scores of 1st, 2nd and 3rd PC for the combined ATR FT-IR spectra of peripheral blood (pink), saliva (blue), semen (light green), urine (yellow) and sweat (light purple).


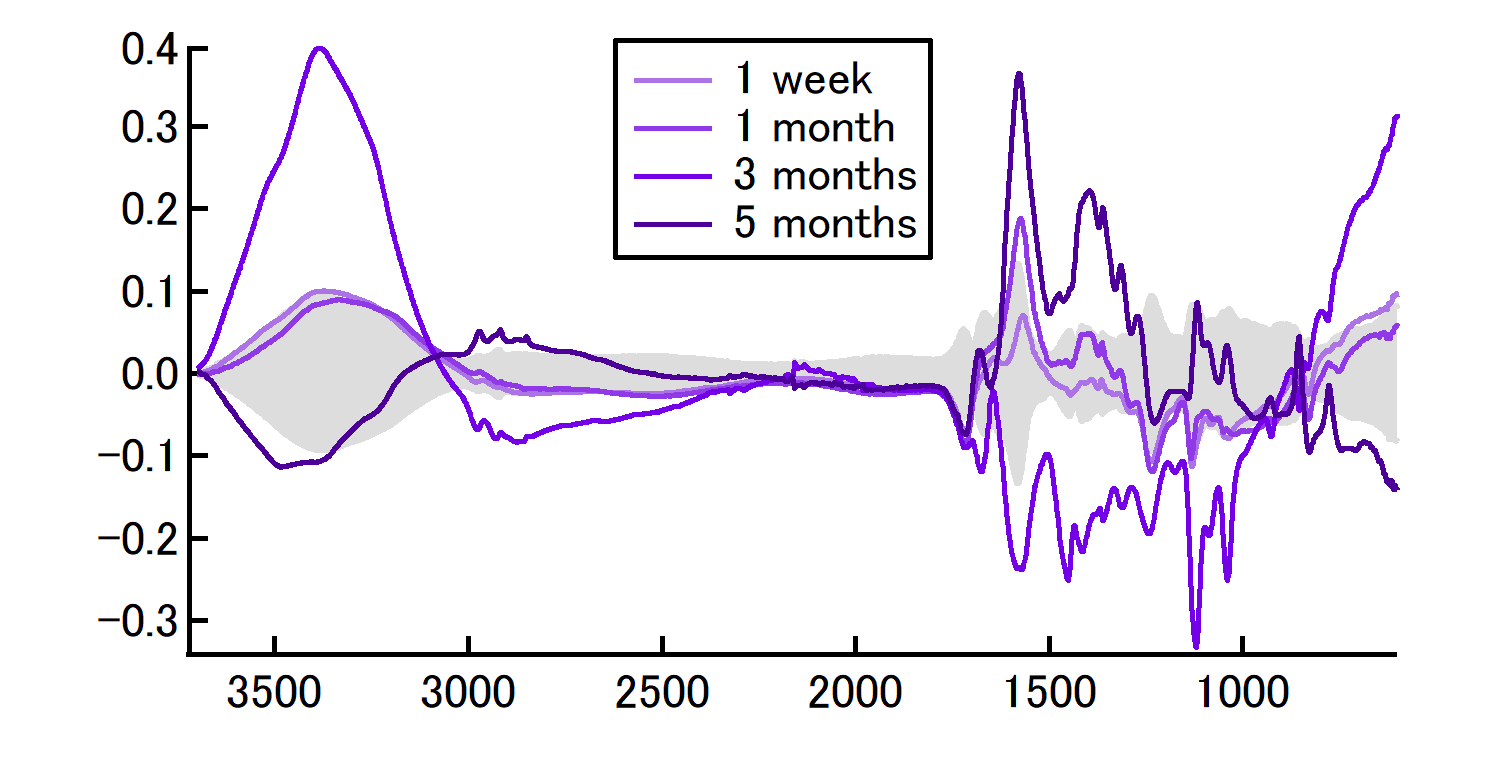

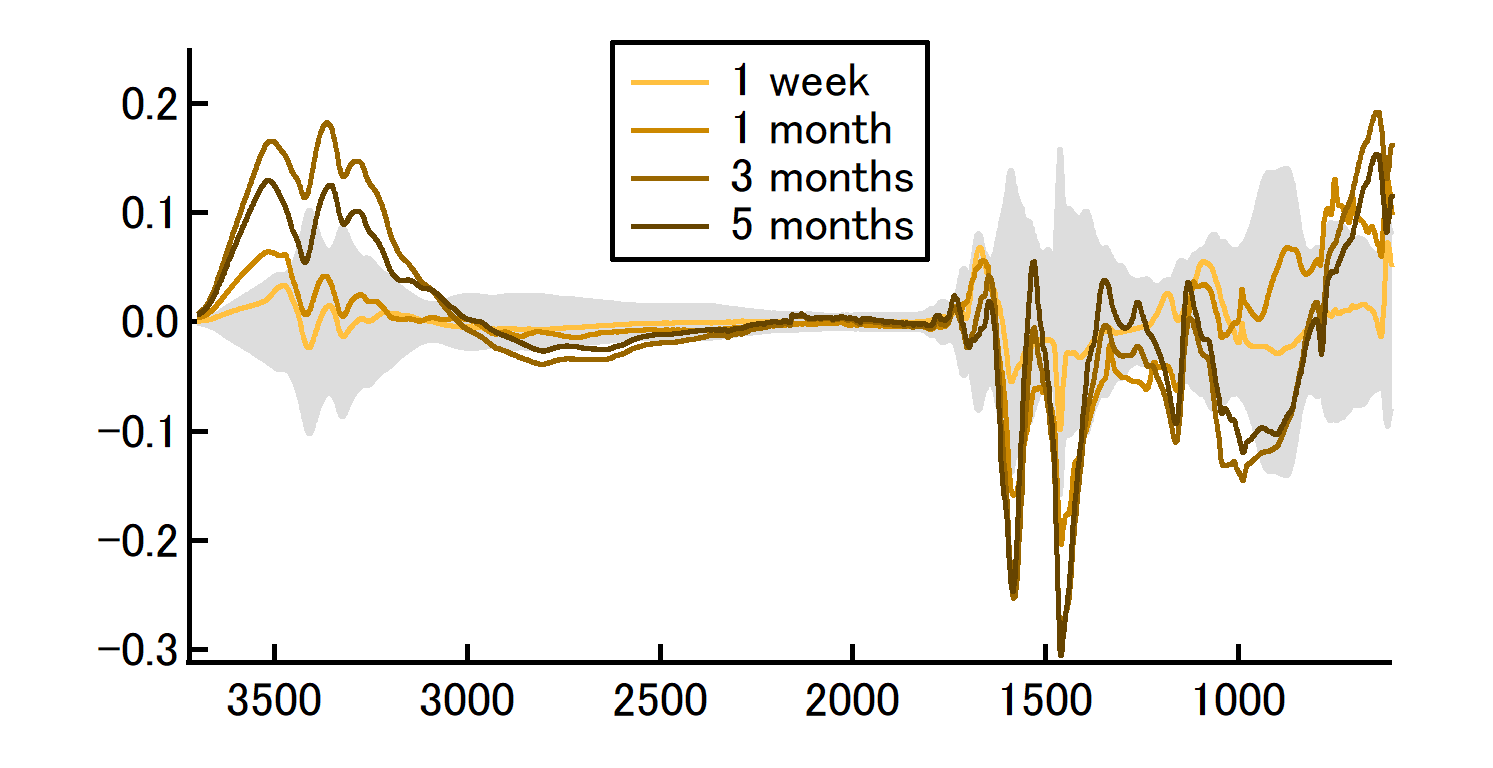

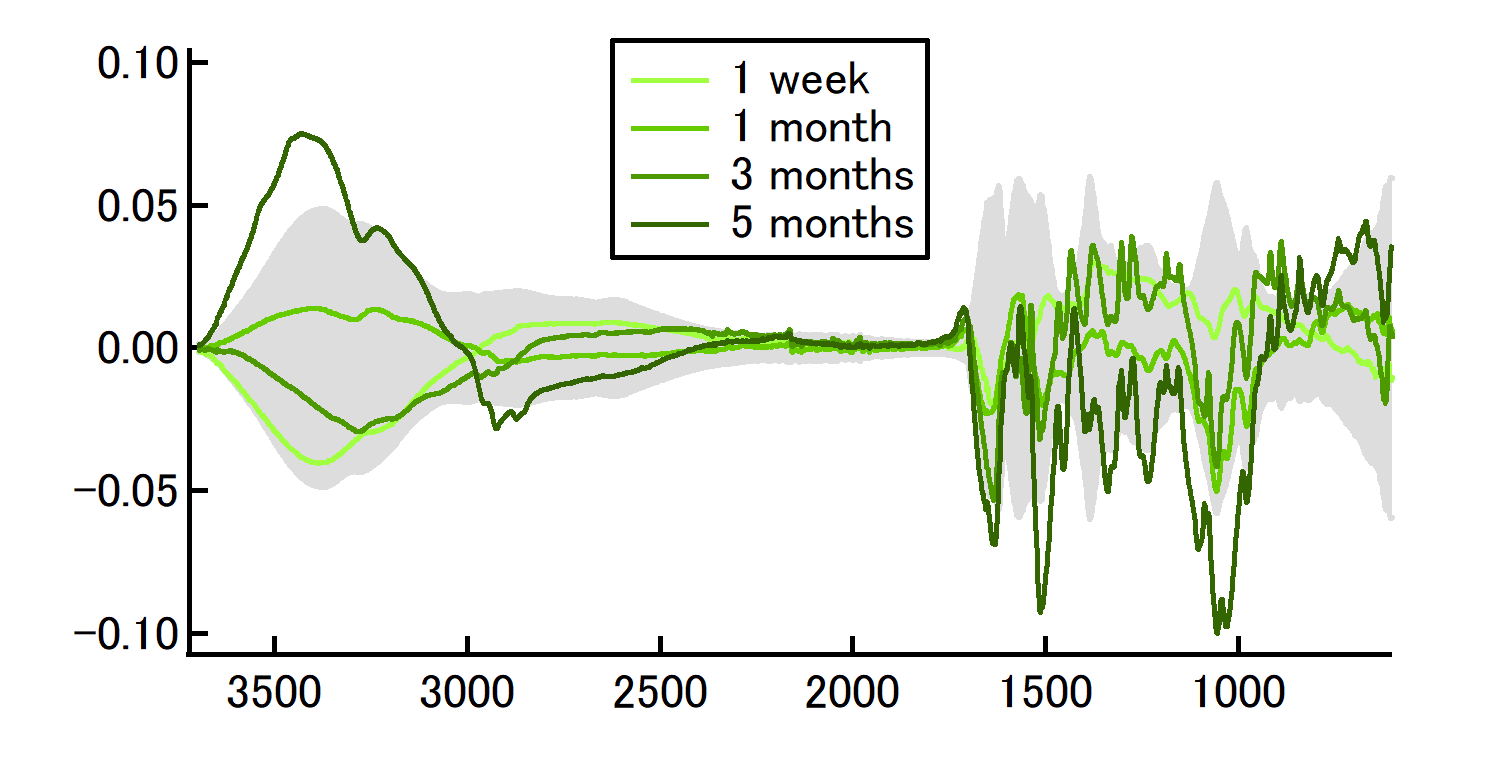

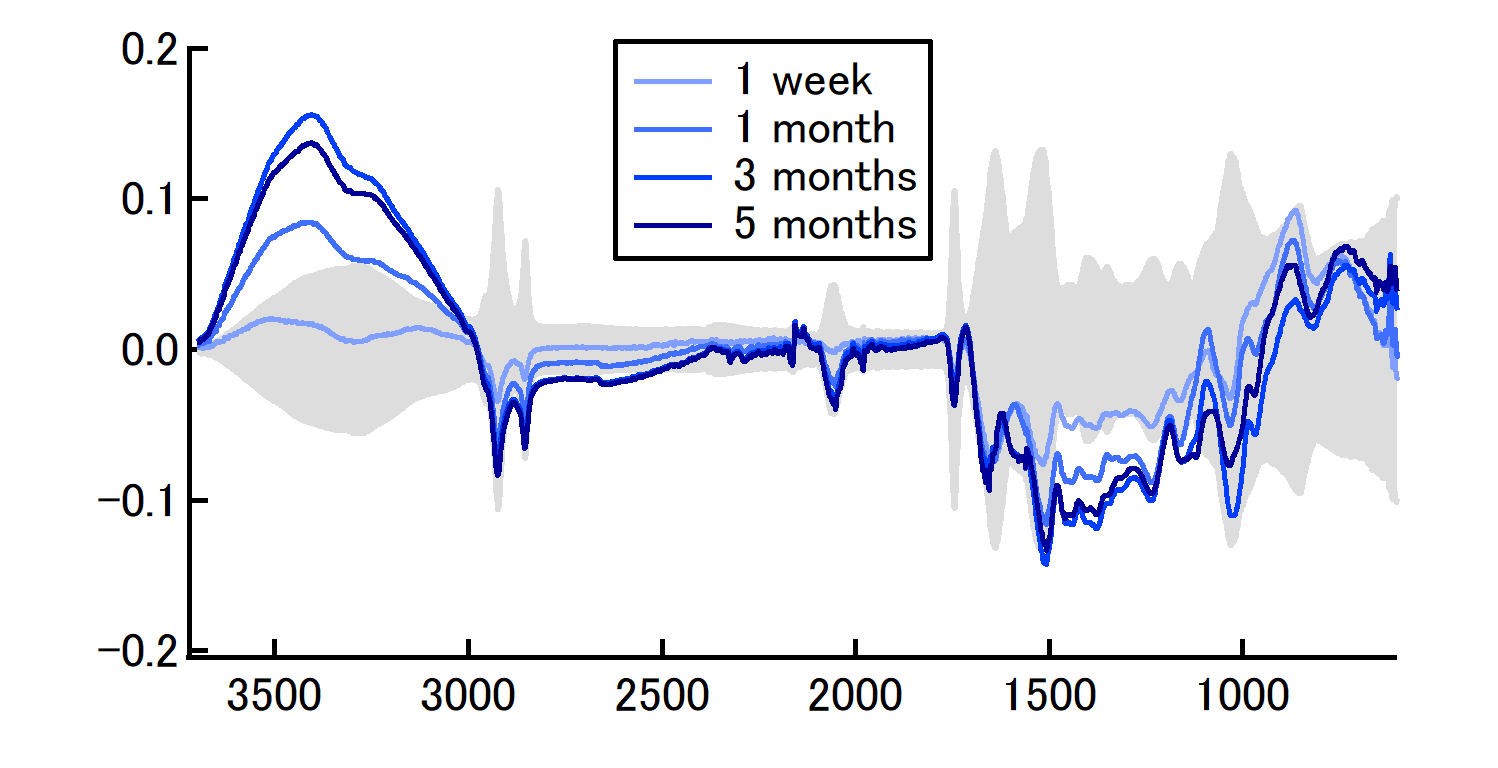

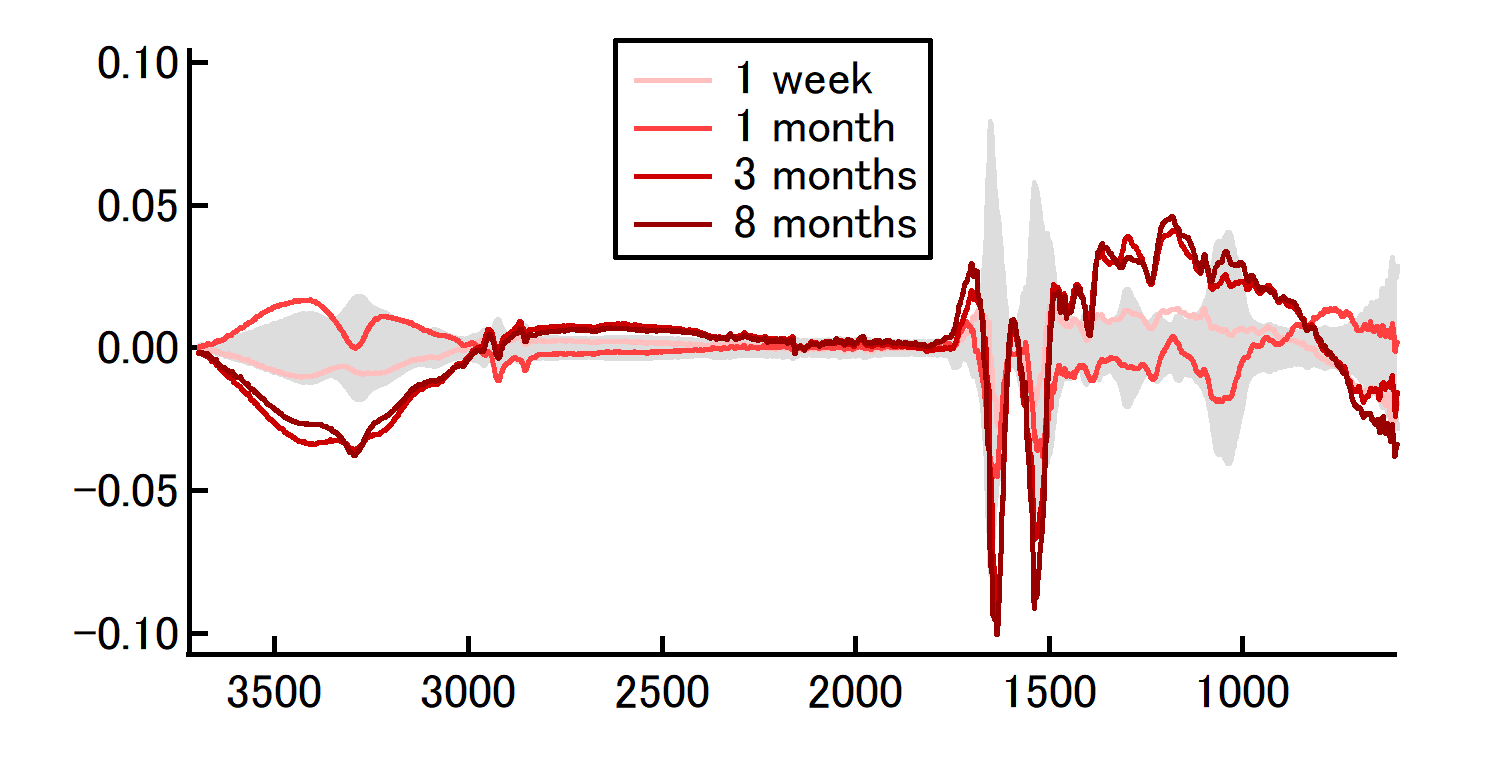


(a)

cm^-1^

Blood

(b)

cm^-1^

Saliva

(c)

cm^-1^

Semen

(d)

cm^-1^

Urine

(e)

cm^-1^

Sweat

**Figure S-3. Difference ATR FT-IR spectra of aged BFs compared to fresh BFs.** Normalized average ATR FT-IR spectra of aged BFs were subtracted by those of 1-day BFs: (a) peripheral blood, (b) saliva, (c) semen, (d) urine and (e) sweat. Gray regions indicate standard deviation spectra of the 1-day BFs.


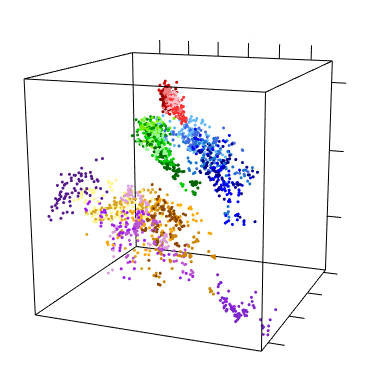


4

2

0

-2

-4

-6

5

-5

0

0

-5

5

10

PC3 (12.0%)

PC2 (29.0%)

PC1 (40.1%)

**Figure S-4. Spectral variances among the ATR FT-IR spectra of aged BFs evaluated by PCA.** A three dimensional dot plot of the calculated scores of 1st, 2nd and 3rd PC for the combined ATR FT-IR spectra of aged BFs, blood (red), saliva (blue), semen (green), urine (yellow) and sweat (purple). The lighter color dots indicate the data of fresher BFs, and the darker colors dots indicate the data of aged BFs.


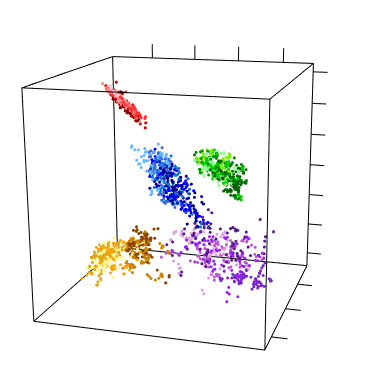


-2

0

2

4

0

2

4

6

-2

-4

-6

-5

0

5


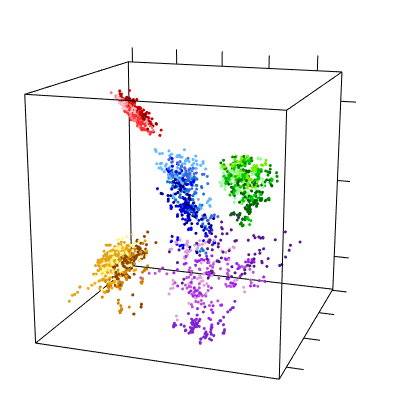


-4

-2

0

2

4

5

0

-5

-5

-10

0

5

(a)

LV1

LV2

LV3

(b)

LV1

LV2

LV3

**Figure S-5. Discrimination of the ATR FT-IR spectra of aged BFs using multi-class classification methods.** Three-dimensional dot plots of the PLS-DA scores for the ATR FT-IR spectra of aged BFs, blood (red), saliva (blue), semen (green), urine (yellow) and sweat (purple), predicted by the 1day-5class PLS-LDA-Q model (a) and the aged-5class PLS-LDA-Q model (b). The lighter color dots indicate the data of fresher BFs, and the darker colors dots indicate the data of aged BFs.

**Table S-1. Discrimination results for the ATR FT-IR spectra of non-BF samples using the PLS-LDA model built with 1-day BF spectra.**

| Non-BF samples | Predicted Body Fluid Type | | | | |
| --- | --- | --- | --- | --- | --- |
|  | Blood | Saliva | Semen | Urine | Sweat |
| Milk | 0 | 4 | 0 | 0 | 1 |
| Soy sauce | 0 | 4 | 0 | 0 | 1 |
| Orange juice | 0 | 3 | 0 | 0 | 2 |
| Ketchup | 0 | 2 | 0 | 0 | 3 |
| Vegetable sauce | 0 | 2 | 0 | 0 | 3 |
| Olive oil | 0 | 1 | 0 | 0 | 4 |
| Mayonnaise | 0 | 1 | 0 | 0 | 4 |
| Shampoo | 0 | 1 | 1 | 0 | 3 |
| Laundry detergent | 0 | 2 | 1 | 0 | 2 |
| Tooth paste | 0 | 2 | 1 | 0 | 2 |
| Glue | 0 | 2 | 1 | 0 | 2 |
| Cotton | 0 | 3 | 1 | 0 | 1 |
| Denim | 0 | 4 | 0 | 0 | 1 |
| polyester | 0 | 4 | 0 | 0 | 1 |

**Table S-2. Discrimination results for the ATR FT-IR spectra of aged BFs (1 day to 8 months) and unexpected samples using the 1day-5class PLS-LDA model.**

| Actual Body Fluid Type | | Predicted Body Fluid Type | | | | |
| --- | --- | --- | --- | --- | --- | --- |
|  |  | Blood | Saliva | Semen | Urine | Sweat |
| Blood | 1 day | 100 | 0 | 0 | 0 | 0 |
|  | 1 week | 100 | 0 | 0 | 0 | 0 |
|  | 1 month | 100 | 0 | 0 | 0 | 0 |
|  | 3 months | 100 | 0 | 0 | 0 | 0 |
|  | 8 months | 100 | 0 | 0 | 0 | 0 |
| Saliva | 1 day | 0 | 90 | 0 | 0 | 0 |
|  | 1 week | 0 | 90 | 0 | 0 | 0 |
|  | 1 month | 0 | 85 | 0 | 0 | 5 |
|  | 3 months | 0 | 75 | 0 | 0 | 15 |
|  | 5 months | 0 | 83 | 0 | 0 | 7 |
| Semen | 1 day | 0 | 0 | 100 | 0 | 0 |
|  | 1 week | 0 | 0 | 100 | 0 | 0 |
|  | 1 month | 0 | 0 | 100 | 0 | 0 |
|  | 3 months | 0 | 0 | 100 | 0 | 0 |
|  | 5 months | 0 | 0 | 100 | 0 | 0 |
| Urine | 1 day | 0 | 0 | 0 | 100 | 0 |
|  | 1 week | 0 | 0 | 0 | 99 | 1 |
|  | 1 month | 0 | 0 | 0 | 93 | 7 |
|  | 3 months | 0 | 0 | 0 | 39 | 61 |
|  | 5 months | 0 | 0 | 0 | 36 | 64 |
| Sweat | 1 day | 0 | 0 | 0 | 0 | 75 |
|  | 1 week | 0 | 0 | 0 | 0 | 75 |
|  | 1 month | 0 | 0 | 0 | 0 | 75 |
|  | 3 months | 0 | 0 | 0 | 0 | 75 |
|  | 5 months | 0 | 0 | 22 | 2 | 51 |

**Table S-3. Discrimination results for the ATR FT-IR spectra of aged BFs (1 day to 8 months) and unexpected samples using the 1day-5class PLS-LDA-Q model.**

| Actual Body Fluid Type | | Predicted Body Fluid Type | | | | | |
| --- | --- | --- | --- | --- | --- | --- | --- |
|  |  | Blood | Saliva | Semen | Urine | Sweat | Outlier*^a^* |
| Blood | 1 day | 99 | 0 | 0 | 0 | 0 | 1 |
|  | 1 week | 97 | 0 | 0 | 0 | 0 | 3 |
|  | 1 month | 99 | 0 | 0 | 0 | 0 | 1 |
|  | 3 months | 68 | 0 | 0 | 0 | 0 | 32 |
|  | 8 months | 76 | 0 | 0 | 0 | 0 | 24 |
| Saliva | 1 day | 0 | 89 | 0 | 0 | 0 | 1 |
|  | 1 week | 0 | 82 | 0 | 0 | 0 | 8 |
|  | 1 month | 0 | 78 | 0 | 0 | 0 | 12 |
|  | 3 months | 0 | 72 | 0 | 0 | 1 | 17 |
|  | 5 months | 0 | 73 | 0 | 0 | 1 | 16 |
| Semen | 1 day | 0 | 0 | 99 | 0 | 0 | 1 |
|  | 1 week | 0 | 0 | 96 | 0 | 0 | 4 |
|  | 1 month | 0 | 0 | 95 | 0 | 0 | 5 |
|  | 3 months | 0 | 0 | 91 | 0 | 0 | 9 |
|  | 5 months | 0 | 0 | 86 | 0 | 0 | 14 |
| Urine | 1 day | 0 | 0 | 0 | 96 | 0 | 4 |
|  | 1 week | 0 | 0 | 0 | 95 | 0 | 5 |
|  | 1 month | 0 | 0 | 0 | 93 | 2 | 5 |
|  | 3 months | 0 | 0 | 0 | 35 | 53 | 12 |
|  | 5 months | 0 | 0 | 0 | 32 | 62 | 6 |
| Sweat | 1 day | 0 | 0 | 0 | 0 | 75 | 0 |
|  | 1 week | 0 | 0 | 0 | 0 | 75 | 0 |
|  | 1 month | 0 | 0 | 0 | 0 | 65 | 10 |
|  | 3 months | 0 | 0 | 0 | 0 | 0 | 75 |
|  | 5 months | 0 | 0 | 0 | 2 | 31 | 42 |
| Non-BF | | 0 | 0 | 0 | 0 | 0 | 70 |

*^a^* Q-tests for each BF group were implemented with a statistical significance level of 99.5%.

**Table S-4. Discrimination results for the ATR FT-IR spectra of aged BFs (1 day to 8 months) and unexpected samples using the aged-5class PLS-LDA-Q model.**

| Actual Body Fluid Type | | Predicted Body Fluid Type | | | | | |
| --- | --- | --- | --- | --- | --- | --- | --- |
|  |  | Blood | Saliva | Semen | Urine | Sweat | Outlier*^a^* |
| Blood | 1 day | 99 | 0 | 0 | 0 | 0 | 1 |
|  | 1 week | 100 | 0 | 0 | 0 | 0 | 0 |
|  | 1 month | 99 | 0 | 0 | 0 | 0 | 1 |
|  | 3 months | 98 | 0 | 0 | 0 | 0 | 2 |
|  | 8 months | 98 | 0 | 0 | 0 | 0 | 2 |
| Saliva | 1 day | 0 | 90 | 0 | 0 | 0 | 0 |
|  | 1 week | 0 | 85 | 0 | 0 | 0 | 5 |
|  | 1 month | 0 | 89 | 0 | 0 | 0 | 1 |
|  | 3 months | 0 | 89 | 0 | 0 | 0 | 1 |
|  | 5 months | 0 | 90 | 0 | 0 | 0 | 0 |
| Semen | 1 day | 0 | 0 | 97 | 0 | 0 | 3 |
|  | 1 week | 0 | 0 | 96 | 0 | 0 | 4 |
|  | 1 month | 0 | 0 | 96 | 0 | 0 | 4 |
|  | 3 months | 0 | 0 | 87 | 0 | 0 | 13 |
|  | 5 months | 0 | 0 | 98 | 0 | 0 | 2 |
| Urine | 1 day | 0 | 0 | 0 | 94 | 0 | 6 |
|  | 1 week | 0 | 0 | 0 | 90 | 0 | 10 |
|  | 1 month | 0 | 0 | 0 | 88 | 0 | 12 |
|  | 3 months | 0 | 0 | 0 | 95 | 0 | 5 |
|  | 5 months | 0 | 0 | 0 | 94 | 0 | 6 |
| Sweat | 1 day | 0 | 0 | 0 | 0 | 74 | 1 |
|  | 1 week | 0 | 0 | 0 | 0 | 75 | 0 |
|  | 1 month | 0 | 0 | 0 | 0 | 69 | 6 |
|  | 3 months | 0 | 0 | 0 | 0 | 75 | 0 |
|  | 5 months | 0 | 0 | 0 | 0 | 66 | 9 |
| Non-BF | | 0 | 0 | 0 | 0 | 0 | 70 |

*^a^* Q-tests for each BF group were implemented with a statistical significance level of 99.0%.
